# Grandmother-inclusive intergenerational approaches: the missing piece of the puzzle for ending FGM/C by 2030?

**DOI:** 10.3389/fsoc.2023.1196068

**Published:** 2023-07-19

**Authors:** Anneke Newman

**Affiliations:** Department of Conflict and Development Studies, Faculty of Political and Social Sciences, Ghent University, Ghent, Belgium

**Keywords:** decolonisation, female genital mutilation/cutting (FGM/C), social norms change, grandmothers, community development, global health, sexual and reproductive health

## Abstract

This brief argues that “grandmother-exclusionary bias” - or the side-lining of female elders as change agents within FGM/C programmes - represents a major obstacle to eradication of these practices. Grandmother-exclusionary bias is prevalent within FGM/C policy and programming. Yet, it goes against evidence of the extensive authority and decision-making roles that grandmothers wield in relation to FGM/C in sub-Saharan Africa, and insights from systems theory and meta-evaluations of FGM/C eradication efforts which stress that sustained change requires engaging those who wield authority over gender and social norms. We use postcolonial and decolonial theory to explain the assumptions about grandmothers which underpin grandmother-exclusionary bias. Finally, we provide recommendations for designing grandmother-inclusive, intergenerational community-led programmes based on a strategy empirically proven to shift social norms underpinning FGM/C.

## 1. Introduction

Female Genital Mutilation/Cutting (FGM/C) is framed within global health discourses as a significant cause and consequence of gendered inequalities. Major financial and material resources have been invested in ending these practices by the international community. In particular, the United Nations Children's Fund (UNICEF) and United Nations Population Fund (UNFPA) wield significant influence over FGM/C discourses because they have invested US$180 million since 2008 in their Joint Programme on the Elimination of Female Genital Mutilation (JPEFGM), which is the largest global programme targeted at the phenomenon (UNFPA, n.d.).

However, this brief's objective is to argue that the dominant approaches used by these organizations to shift gender and social norms underpinning FGM/C in the Global South risk conflicting with authority structures, resulting in low efficacy and high possibilities of community resistance. We show that this evident in “grandmother-exclusionary bias,” or the exclusion of grandmothers/female elders as change agents in favor of youth, women of reproductive age, men and boys, and male traditional/religious leaders. Our evidence for grandmother-exclusionary bias derives from a critical discourse analysis of seven reports published between 2015 and 2021 by the UNFPA and UNICEF in relation to the JPEFGM, which made recommendations of which community members to engage as change agents in FGM/C programmes (Newman, 2023). Based on a review of secondary literature on health-related behavior strategies, we propose that grandmother-exclusionary bias reflects coloniality of knowledge in global health and gender and development, which is when Euro-North-American-centric cultural norms underpin academic research, theory, policy, and intervention strategies (Khan et al., 2021; Aloudat, 2022).

In contrast, we present evidence on trends in authority and decision-making that underpin FGM/C in sub-Saharan Africa, results of meta-evaluations of FGM/C eradication programmes, and insights from systems theory approaches to health-related behavior change, to argue that community-based FGM/C social norms change programmes are more likely to be effective if they engage grandmothers as a priority group within an intergenerational strategy *under the right conditions*. Finally, we provide recommendations for policy-makers and practitioners on developing and implementing grandmother-inclusive intergenerational approaches in Africa based on the Girls Holistic Development programme of NGO The Grandmother Project-Change Through Culture, which independent evaluations (Shaw et al., 2020; Kohli et al., 2021) prove has shifted gender and social norms underpinning FGM/C in southern Senegal.

## 2. The international policy context: social norms change and FGM/C

For 15 years, the main theoretical framework used for addressing FGM/C has been that of social norms change (Cislaghi and Heise, 2018). There are differences in how social norms change has been defined and operationalized across disciplines, but in global health the dominant model draws largely from psychology and equates social norms with social expectations (Edberg and Krieger, 2020). Social norms are defined as informal rules of behavior that dictate what is acceptable in a given context (Chung and Rimal, 2016; Cislaghi and Heise, 2018). Internalized by individuals, social norms are posited as regulating behavior through perceived consensus, reward for conformity, and sanctions for non-conformity. To shift social norms which underpin FGM/C, programmes focus on changing people's expectations about the likelihood of sanctions or rewards (Mackie et al., 2015).

Common activities used to shift norms underpinning FGM/C include: exposing community members to “positive deviants” who defy dominant norms; facilitating discussions with community members to challenge norms, or reveal that not everyone actually follows them; promoting public declarations assumed to attest to a collective change in behavior; encouraging sanctions on harmful norms; and supporting leaders to denounce such norms (Bicchieri and Mercier, 2014). These activities are used and endorsed by the major donors, international organizations and NGOs working on FGM/C, including UNICEF, UNFPA and their partners.

## 3. Critical analysis of policy: grandmother-exclusionary bias

The dominant strategies used to shift social norms underpinning FGM/C risk being weakly effective and likely to cause backlash because the choice of actors typically solicited to act as change agents conflicts with authority and decision-making dynamics within FGM/C-practicing communities (Shell-Duncan et al., 2018). UNFPA, UNICEF and their partners prioritize activities with adolescent girls, followed by women of reproductive age, customary cutters, men and boys, and male religious/traditional leaders. Meanwhile, grandmothers or female elders are hardly mentioned. If they are, they are framed as less relevant than other actors, or as a harmful rather than potentially progressive force (Newman, 2023).

In contrast, qualitative evidence on the influence of extended family members on FGM/C from contexts as diverse as Ethiopia (Abathun et al., 2016), Eritrea (Alhassan et al., 2016), the Gambia (Ahmadu, 2005), Guinea Bissau (Alhassan et al., 2016), Mali (Gosselin, 2000; Leon-Himmelstine et al., 2022), Senegal (Alhassan et al., 2016; Shell-Duncan et al., 2018), Sierra Leone (Ahmadu, 2005), Somalia (Gele et al., 2012; Abubakar, 2013), and the Sudan (Boddy, 1982) show that grandmothers/female elders often wield significant authority over FGM/C individually and collectively as family decision-makers, social norms influencers, and executors of the surgeries and/or accompanying rituals. Within their families, grandmothers' authority can equal or exceed that of parents, including fathers, especially when mothers are young (in their teens or early twenties). These studies also show that, in these contexts, it is often considered disrespectful for men or youth to publicly disagree with, or oppose, female elders' decisions relating to FGM/C.

This situation is not surprising as, in sub-Saharan Africa, where most FGM/C takes place, societies historically exhibit “dual-sex institutions” where men and women are framed as separate but complementary in characteristics, and are associated with different activities and spheres of influence (Nnaemeka, 2005). In such societies, gender intersects with hierarchies based on seniority, where biological age and position in kinship structures confers respect and authority (Amadiume, 1987; Oyěwùmí, 1997). Seniority and dual-sex institutions mean that older women—rather than men—often exert principal authority in decision-making in spheres coded as feminine, including in relation to children's health, and women's sexual and reproductive health (SRH) (Aubel, 2021; Aubel et al., 2021). While such norms are changing, especially in urban contexts, middle-class nuclear families, or diaspora communities, recent evidence that extended families, including grandmothers, from Senegambia and Guinea Bissau influence parents in Europe (Alhassan et al., 2016) suggests that grandmothers' influence over FGM/C should be assumed at the outset and disproved—rather than the opposite, which is currently the case.

Meanwhile, meta-evaluations of FGM/C programmes suggest that, to be successful, activities must engage the people who wield authority over relevant social norms (Muteshi and Sass, 2005; Denison et al., 2009; Johansen et al., 2013) as they can more successfully foster conformity to their example, and play a leadership role in their social setting (Newman, 2023). Meta-evaluation findings align with insights from systems approaches to health-related behavior change, which show that community-based programmes can only bring about sustained change if people with authority support that change (Figueroa et al., 2002; Foster-Fishman et al., 2007). Given the wealth of data on grandmothers' decision-making authority in relation to FGM/C, and evidence that they can be progressive change agents (Shell-Duncan et al., 2018; Newman, 2023), grandmother-exclusionary bias among the dominant organizations working to eradicate FGM/C requires an explanation.

## 4. Theoretical critique: postcolonial and decolonial theory

We argue that youth-focused programmes and grandmother-exclusionary bias reflect several assumptions underpinned by coloniality of knowledge, namely when Euro-North-American-centric cultural norms shape academic research, theory, policy decisions, and intervention strategies (Khan et al., 2021; Aloudat, 2022; Newman, 2023).

First, the focus on adolescent girls as change agents within FGM/C programmes has several explanations. It reflects the problematic tendency of public health interventions to focus on “at risk” groups, rather than wider sections of the population, or structural factors, which contribute to these risks (Whitehead, 2007). The emphasis on adolescent girls also reflects the “Girl Effect” discourse which has dominated in gender and development since the mid-2000s, based on the idea that “girl power” is the best way to counter “harmful traditional practices” (Koffman and Gill, 2013). Hence, the main strategy used to counter gender inequalities has been girls' empowerment approaches (GEAs) which entail providing female adolescents with information, skills and networks to resist practices deemed harmful (Lee-Rife et al., 2012).

Despite their popularity, Girl Effect discourse and GEAs have been strongly critiqued by postcolonial scholars. They are based on homogenizing representations of “Third World girls” oppressed by “culture” and “religion”—understood in simplistic terms as fixed sets of values, behaviors, and traditions inherited from past generations. Girls are framed as progressive, while their families are depicted as sites of harm, fathers and husbands as malicious patriarchs, and older women as conservative victims of false consciousness (Koffman and Gill, 2013). According to these logics, the solution to problems posed by “culture” or “tradition” must come from outside, with girls encouraged to assume Western values of individualism, independence, assertiveness and combativeness, through allying with NGO staff, social workers, police, or teachers, in opposition to their families (Koffman and Gill, 2013; Bessa, 2020). GEAs have also been critiqued by meta-evaluations for being weakly effective, as they tend to increase girls' knowledge and confidence but not authority—referred to as “informed powerlessness” (Bessa, 2020)—and thus have a limited impact on gender and social norms. Furthermore, openly resisting and confronting their families can render girls isolated and vulnerable, and failure to create community-level consensus to shift norms risks pushing harmful practices underground (Bessa, 2020). Finally, because FGM/C is typically conducted by adults concerned with acting in girls' best interests given their socio-cultural context, framing family members solely as being “the problem” risks creating backlash among parents and elders (Esho et al., 2011).

Given the limitations of GEAs, “community-based” approaches have been promoted instead (Warner et al., 2014). This explains the more recent tendency of FGM/C programmes to engage men and boys, and male community and religious leaders. However, this choice of actors as change agents over grandmothers is also based on flawed assumptions. Either it presumes that grandmothers wield minimal decision-making authority, which reflects Western second-wave feminist assumptions about gender relations grounded in models of patriarchy that entail men dominating women within a simplistic gender binary (Mama, 2005; Arnfred, 2014). This assumption ignores the empirical evidence cited above, and work of African gender scholars (Amadiume, 1987; Oyěwùmí, 1997), who argue that, across the continent, seniority intersects with gender to confer authority on female elders in relation to SRH.

Alternatively, grandmother-exclusionary bias is based on assumptions that grandmothers do influence FGM/C, but that they invariably support the practices and are incapable of changing their views. This ignores growing evidence that grandmothers do not necessarily support FGM/C; are perfectly able to change their views; and can become influential change agents if engaged respectfully in culturally-appropriate ways (Shell-Duncan et al., 2018; Newman, 2023). Indeed, the assumption of grandmothers' conservatism reflects Euro-North-American sexist ageism - which is also central to the Girl Effect discourse and GEAs - that presents girls as having change-making potential, but older women as obstacles to change (Koffman and Gill, 2013). In contrast, postcolonial African feminists/womanists have long argued that, while seniority can constrain younger women, intergenerational relationships also have enabling potential (Nnaemeka, 2005; Arnfred, 2014). By pitting girls and older women against each other, Girl Effect discourse and GEAs ignore their shared vulnerabilities, and possibilities of solidarity and collective action (Koffman and Gill, 2013; Bessa, 2020). Feminists from the Global North have also argued that grandmother-exclusionary bias is systemic within development generally, as privilege is given to girls, adolescents and young adults—while grandmothers or older women are rarely mentioned. If they are, it is as people with vulnerabilities or as passive recipients of care, rather than as change agents (Lipman, 2013; Sleap et al., 2013; Varley, 2013). Finally, organizations frequently engage other categories of people who tend to support FGM/C at the outset—like male religious leaders—so to not offer grandmothers the same opportunity to learn and change reflects a sexist double standard (Newman, 2023).

Finally, grandmother-exclusionary bias often has unintended negative consequences. Programmes which privilege men and boys over grandmothers risk making women vulnerable because they “weaken female power centers within society and bring women's bodies and lives under the hegemonic control and management of local male religious or political leaders” (The Public Policy Advisory Network on Female Genital Surgeries in Africa, 2012; p. 23). Strategies which pit youth against elders also undermine intergenerational trust and communication, social cohesion, transmission of indigenous knowledge, and communities' internal capacity to mobilize collectively to address their own problems—further increasing dependency on NGOs (Newman, 2023). In contrast, a collective of health scientists and anthropologists called The Public Policy Advisory Network on Female Genital Surgeries in Africa have demanded that women—especially older women—be centered within change processes (2012).

## 5. Grandmother-inclusive cultural renewal: a decolonial paradigm for FGM/C programmes

Effective grandmother-inclusive intergenerational strategies which shift FGM/C social norms do exist—for instance, The Grandmother Project-Change through Culture's (GMP) Girls Holistic Development (GHD) programme in Vélingara, Senegal. Rigorous evidence shows that the GHD strategy has shifted social norms underpinning FGM/C, as well as child marriage and premature school-leaving. In 2015, GHD was identified by the United States Agency for International Development (USAID)-funded Passages project based at the Institute of Reproductive Health (IRH) at Georgetown University as a promising example of a community-level programme that shifted norms related to SRH. The subsequent IRH realist evaluation of the GHD programme (IRH, 2019; Shaw et al., 2020; Kohli et al., 2021) confirmed that GMP contributed to shifting culturally-embedded social norms and practices by empowering grandmothers—in collaboration with other influential community actors—to create an enabling environment around adolescent girls. We therefore use the GHD model to suggest conditions required for working collaboratively with grandmothers to shift FGM/C gender and social norms.

GMP's approach works not only because it engages grandmothers as change agents: the terms of their inclusion are also crucial. Dominant FGM/C social norms change programmes often frame local cultural institutions and values as obstacles to progressive change (and by extension grandmothers, who are typically custodians of tradition), which often causes backlash. Instead, GMP sees culture—including knowledges, values, customary roles, authority structures, and leadership—as a dynamic set of resources which can support progressive change. GMP's strategy draws inspiration from the cultural renewal approach where “by focusing on participatory involvement of local peoples in the process, cultural practices [can] be reformulated and renewed.” Hence, progressive aspects of culture are reinforced, and harmful elements discarded (White and Nair, 1994; p. 139).

To catalyze cultural renewal in Senegal, GMP adapted the systems theory-informed Communication For Social Change (CFSC) methodology developed by community development experts at the Bloomberg School of Public Health at Johns Hopkins University (Figueroa et al., 2002). This methodology uses facilitated dialogue to build consensus in favor of change among community authorities who shape gender and social norms relating to SRH—with grandmothers playing a leading role, alongside other key influencers. Despite mobilizing traditional authorities for change, this approach also tackles customary gender norms which marginalize girls and young women in decision-making processes. Therefore, it also involves reducing hierarchies between people on the basis of age and gender, and facilitating equitable dialogue to ensure that the interests of the marginalized take center stage.

GMP's approach also avoids directive messaging telling people what to do. Instead, it uses transformative Paulo Freirean adult education methods to promote collective reflection and critique of deeply rooted practices like FGM/C and child marriage, as well as hierarchical modes of decision-making. It supports the building of intergenerational collaborations between grandmothers and adolescent girls and builds their collective capacity to mobilize to protect and promote the interests of girls at family and community level (Musoko et al., 2012; GMP, 2021). This approach aligns with African feminisms/womanisms that value conciliation, consensus, intergenerational solidarity between women, and community cohesion (Nnaemeka, 2005).

## 6. Recommendations for community-led intergenerational grandmother-inclusive FGM/C programmes

These recommendations are intended for policymakers and practitioners working at community level to shift social norms underpinning FGM/C.

### 6.1. Build a solid evidence base

- Using quantitative and qualitative methods, identify context-specific drivers of FGM/C; individuals' knowledge, attitudes, and dominant social and gender norms—including what people have to gain from norms, and who enforces them and how—which differentiates respondents' answers according to seniority and gender;
- Identify the main context-specific decision-makers, social norms influencers, and executors relevant to FGM/C within the extended family and community, and their respective degrees of authority;
- Investigate family and community decision-making dynamics, and how different actors' attitudes and authority intersect to produce FGM/C trends.
- Develop and operationalize MERL (monitoring, evaluation, research, learning) systems at all programme stages which differentiate between changes in *knowledge, attitudes, social and gender norms*, and *practices* to understand which activities have catalyzed change and how.

### 6.2. Use systems change approaches to catalyze consensus on community-directed change

- Understand the principles of systems theory (also known as systems science, or systems change) and community development before designing programmes;
- Work directly with a wide range of community groups, particularly those categories of persons who wield most authority over FGM/C;
- Respect the cultural roles, experience, and knowledge of elders—especially grandmothers—and involve them explicitly and respectfully in activities, while simultaneously designing activities to amplify the voices, and increase the authority, of groups affected by FGM/C but who have limited decision-making authority;
- For each community group by age and gender, identify key individuals and role models who are influential and can mobilize other members of their peer group (i.e. formal and informal leaders);
- Facilitate non-directional inter-generational dialogue between elders and youth to share knowledge and experiences; define problems, priorities and solutions; and reach a community-wide consensus in favor of change.

## 7. Conclusion

Grandmothers are among the principal authorities who influence gender and social norms underpinning FGM/C in sub-Saharan Africa. Insights from systems theory and meta-evaluations of FGM/C programmes show that social norms change strategies can only work if they engage the people who wield authority over those norms. Yet, organizations working to end FGM/C often display grandmother-exclusionary bias by side-lining grandmothers as change agents. Their focus on adolescent girls, men and boys, and male religious/community leaders reflects Euro-North-American-centric assumptions about gendered decision-making, authority, and attitudes which do not align with trends within extended families in African contexts. These organizations also endorse approaches which risk causing unintended negative consequences by undermining intergenerational relationships and social cohesion, and hence provoke community backlash. In contrast, this paper draws on the gender and social norms change strategy of NGO The Grandmother Project-Change Through Culture to propose recommendations for grandmother-inclusive intergenerational programmes which use facilitated dialogue to catalyze consensus among community authorities in favor of collective change which favors the interests of the marginalized.

## Author contributions

The author confirms being the sole contributor of this work and has approved it for publication.

## References

[B1] Abathun A. D. Sundby J. A. Gele A. A. (2016). Attitude toward female genital mutilation among Somali and Harari people, Eastern Ethiopia. Int. J. Women Health. 8, 557–569. 10.2147/IJWH.S112226 27785105 PMC5065096

[B2] Abubakar N. (2013). Global discourse, local practice: female circumcision and inter-generational conflict in a Somali diaspora community. Perspect. Glob. Dev. Technol. 12, 476–488. 10.1163/15691497-12341268

[B3] Ahmadu F. S. (2005). Cutting the anthill: the symbolic foundations of female and male circumcision rituals among the Mandinka of Brikama, the Gambia (PhD thesis). Department of Anthropology, School of Economics, London, United Kingdom.

[B4] Alhassan Y. N. Barrett H. Brown K. E. Kwah K. (2016). Belief systems enforcing female genital mutilation in Europe. Int. J. Hum. Rights Healthc. 9, 29–40. 10.1108/IJHRH-05-2015-001524308471

[B5] Aloudat T. (2022). Can the sick speak? Global health governance and health subalternity. Soc. Sci. 11, 417. 10.3390./socsci11090417

[B6] Amadiume I. (1987). Male Daughters, Female Husbands: Gender and Sex in an African Society. London: Zed Press.

[B7] Arnfred S. (2014). Battlefields of Knowledge: Conceptions of Gender in Development Discourse. In Africa-Centred Knowledges: Crossing Fields and Worlds, edited by Brenda Cooper and Robert Morrell, 51–63. Woodbridge: James Currey.

[B8] Aubel J. (2021). Grandmothers — a neglected family resource for saving newborn lives. BMJ Global Health 6, 003808. 10.1136/bmjgh-2020-003808 33589417 PMC7887373

[B9] Aubel J. Martin S. L. Cunningham K. (2021) Introduction: A family systems approach to promote maternal, child adolescent nutrition. Matern. Child Nutr. 17, 1–9. 10.1136/bmjgh-2022-009914 34241950 PMC8269145

[B10] Bessa T. (2020). Informed powerlessness: child marriage interventions and third world girlhood discourses. Third World Quart. 40, 1941–1956. 10.1080/01436597.2019.1626229

[B11] Bicchieri C. Mercier H. (2014). “Norms and beliefs: how change occurs,” in The Complexity of Social Norms, eds M. Xenitidou and B. Edmonds (New York, NY: Springer), pp. 37–54.

[B12] Boddy J. (1982). Womb as oasis: the symbolic context of pharaonic circumcision in rural northern Sudan. Am. Ethnol. 9, 682–698. 10.1525/ae.1982.9.4.02a00040

[B13] Chung A. Rimal N. R. (2016). Social norms: a review. Rev. Commun. Res. 4, 1–28. Available online at: https://www.ssoar.info/ssoar/handle/document/45755

[B14] Cislaghi B. Heise L. (2018). Using social norms theory for health promotion in low income countries. Health Promot. Int. 3, 1–8. 10.1093./heapro/day017 29579194 PMC6662293

[B15] Denison E. Berg R. C. Lewin S. Fretheim A. (2009). Effectiveness of Interventions Designed to Reduce the Prevalence of Female Genital Mutilation/Cutting. Oslo: Nasjonalt kunnskapssenter for helsetjenesten. 29320117

[B16] Edberg M. Krieger L. (2020). Recontextualizing the social norms construct as applied to health promotion. SSM Pop. Health 10, 1–9. 10.1016/j.ssmph.2020.100560 32140543 PMC7047191

[B17] Esho T. Van Wolputte S. Enzlin P. (2011). The socio-cultural-symbolic nexus in the perpetuation of female genital cutting: a critical review of existing discourses. Afrika Foc. 24, 53–70. 10.21825/af.v24i2.4997

[B18] Figueroa M. E. K. Rani D. L. Manju Lewisnline G. (2002). Communication for Social Change: An Integrated Model for Measuring the Process and Its Outcomes. New York., NY: The Rockefeller Foundation and Johns Hopkins University Center for Communication Programs.

[B19] Foster-Fishman P. G. Nowell B. Yang H. (2007). Putting the system back into systems change: a framework for understanding and changing organizational and community systems. Am. J. Commun. Psychol. 39, 197–215. 10.1007/s10464-007-9109-0 17510791

[B20] Gele A. A. Kumar B. Hjelde K. H. Sundby J. (2012). Attitudes toward female circumcision among Somali immigrants in Oslo: a qualitative study. Int. J. Womens Health 4, 7–17. 10.2147/IJWH.S27577 22312195 PMC3271810

[B21] GMP. (2021). Catalyzing Change for Girls: Grandmothers Support Girls' Holistic Development. Summary of Approach and Achievements. Dakar: The Grandmother Project-Change Through Culture.

[B22] Gosselin C. (2000). Feminism, anthropology and the politics of excision in mali: global and local debates in a postcolonial world. Anthropologica 42, 43–60. 10.2307/25605957

[B23] IRH. (2019). Grandmother Project - Change through Culture: Program for Girls' Holistic Development: Qualitative Research Report. Washington, DC: Institute of Reproductive Health, Georgetown University for the American Agency for International Development (USAID).

[B24] Johansen R. E. B. Diop N. J. Laverack G. Leye E. (2013). What works and what does not: a discussion of popular approaches for the abandonment of female genital mutilation. Obstet. Gynecol. Int. 3, 1–10. 10.1155./2013/348248 23737795 PMC3655658

[B25] Khan M. Abimbola S. Aloudat T. Capobianco E. Hawkes S. Rahman-Shepherd A.. (2021). Decolonising global health in 2021: a roadmap to move from rhetoric to reform. BMJ Glob. Health. 4, 5604. 10.1136./bmjgh-2021-005604 33758016 PMC7993212

[B26] Koffman O. Gill R. (2013). ‘The revolution will be led by a 12-year-old girl': girl power and global biopolitics. Fem. Rev. 105, 83–102. 10.1057/fr.2013.16

[B27] Kohli A. Shaw B. Guntzberger M. Aubel J. Coulibaly M. Igras S.. (2021). Transforming social norms to improve girl-child health and wellbeing: a realist evaluation of the girls' holistic development program in rural senegal. Reprod. Health 18, 1–14. 10.1186/s12978-021-01295-5 34861876 PMC8641196

[B28] Lee-Rife S. Malhotra A. Warner A. Glinski A. M. (2012). What works to prevent child marriage: a review of the evidence. Stud. Fam. Plan. 43, 287–303. 10.1111/j.1728-4465.2012.00327.x 23239248

[B29] Leon-Himmelstine C. Salomon H. Diarra A. Samman E. (2022). ‘*A cut woman is the pride of all her relatives': A situation analysis of female genital mutilation in Mali. Executive Summary*. London: Overseas Development Institute.

[B30] Lipman V. (2013). The Invisibility of Older Women in International Development. In A Compendium of Essays: Has the Sisterhood Forgotten Older Women? edited by Sally-Marie Bamford and Jessica Watson. London: International Longevity Centre UK, pp. 111–13.

[B31] Mackie G. Moneti F. Shakya H. Denny E. (2015). What Are Social Norms? How Are They Measured? San Diego: University of California (Center on Global Justice, for UNICEF).

[B32] Mama A. (2005). Gender Studies for Africa's Transformation. In African Intellectuals: Rethinking Politics, Language, Gender, and Development, edited by Thandika Mkandawire, 94–116. London: Zed Books and CODESRIA.

[B33] Musoko A. Saou Scoppa C. Manoncourt E. Aubel J. (2012). Girls and Grandmothers Hand-in-hand: Dialogue Between Generations for Community Change. Rome: World Vision and Grandmother Project.

[B34] Muteshi J. Sass J. (2005). Female Genital Mutilation in Africa: An Analysis of Current Abandonment Approaches. Nairobi: PATH.

[B35] Newman A. (2023). Decolonising social norms change: from ‘grandmother-exclusive bias' to ‘grandmother-inclusive' approaches. Third World Quart. 4, 8408. 10.1080./01436597.2023.2178408

[B36] Nnaemeka Ob. (2005). “*Mapping African Feminisms”: Adapted Version of “Introduction: Reading the Rainbow”, from Sisterhood, Feminisms and Power: From Africa to the Diaspora. In Readings in Gender in Africa, edited by Andrea Cornwall,* 31–40. *Bloomington and Indianapolis, IN*. Oxford: Indiana University Press and James Currey.

[B37] Oyěwùmí O. (1997). The Invention of Women: Making an African Sense of Western Gender Discourse. Minneapolis, MN: University of Minnesota Press.

[B38] Shaw B. Kohli A. Igras S. (2020). Grandmother Project – Change through Culture: Girls' Holistic Development Program Quantitative Research Report. Washington, DC: Institute for Reproductive Health, Georgetown University with the United States Agency for International Development (USAID).

[B39] Shell-Duncan B. Moreau A. Wander K. Smith S. (2018). The role of older women in contesting norms associated with female genital mutilation/ cutting in senegambia: a factorial focus group analysis. PLOS ONE 13, 9217. 10.1371./journal.pone.0199217 30044770 PMC6059403

[B40] Sleap B. Eppu M. J. Mark G. (2013). :Older women in low-and middle-income countries – forgotten by feminism?” in *A Compendium of Essays: Has the Sisterhood Forgotten Older Women?* edited by Sally-Marie Bamford and Jessica Watson, 107–10. London: International Longevity Centre UK.

[B41] The Public Policy Advisory Network on Female Genital Surgeries in Africa. (2012). Seven things to know about female genital surgeries in Africa. Hastings Center Report 6, 19–27. 10.1002/hast.81 23138367

[B42] UNFPA (n.d.) UNFPA-UNICEF Joint Programme on the Elimination of Female Genital Mutilation unfpa.org. Available online at: https://www.unfpa.org/unfpa-unicef-joint-programme-female-genital-mutilation (accessed March 23 2023).

[B43] Varley A. (2013). Feminism's Pale Shadows: Older Women, Gender and Development. In A Compendium of Essays: Has the Sisterhood Forgotten Older Women? edited by Sally-Marie Bamford and Jessica Watson, 114–16. London: International Longevity Centre UK.

[B44] Warner A. Stoebenau K. Glinski A. M. (2014). More Power to Her: How Empowering Girls can Help End Child Marriage. Washington, DC: International Center for Research on Women.

[B45] White S. A. Nair S. K. (1994). “Participatory development communication as cultural renewal,” in Participatory Communication: Working for Change and Development, eds Shirley A. White, Sadanandan K. Nair, and Joseph Ascroft (London: Sage Publications), pp. 138–93.

[B46] Whitehead M. (2007). A Typology of Actions to Tackle Social Inequalities in Health. J. Epidemiol. Commun. Health 61, 473–78. 10.1136/jech.2005.037242 17496254 PMC2465710

